# Viral Transduction of Human Rod Opsin or Channelrhodopsin Variants to Mouse ON Bipolar Cells Does Not Impact Retinal Anatomy or Cause Measurable Death in the Targeted Cells

**DOI:** 10.3390/ijms222313111

**Published:** 2021-12-03

**Authors:** Phillip Wright, Jessica Rodgers, Jonathan Wynne, Paul N. Bishop, Robert J. Lucas, Nina Milosavljevic

**Affiliations:** Faculty of Biology Medicine and Health, University of Manchester, Manchester M13 9PL, UK; phill_wright90@hotmail.co.uk (P.W.); jessica.rodgers@manchester.ac.uk (J.R.); Jonathan.Wynne@manchester.ac.uk (J.W.); paul.n.bishop@manchester.ac.uk (P.N.B.); Robert.Lucas@manchester.ac.uk (R.J.L.)

**Keywords:** optogenetics, vision restoration, ON bipolar cells, rodopsin, channelrhodopsin

## Abstract

The viral gene delivery of optogenetic actuators to the surviving inner retina has been proposed as a strategy for restoring vision in advanced retinal degeneration. We investigated the safety of ectopic expression of human rod opsin (hRHO), and two channelrhodopsins (enhanced sensitivity CoChR-3M and red-shifted ReaChR) by viral gene delivery in ON bipolar cells of the mouse retina. Adult *Grm6^Cre^* mice were bred to be retinally degenerate or non-retinally degenerate (homozygous and heterozygous for the *rd1^Pde6b^* mutation, respectively) and intravitreally injected with recombinant adeno-associated virus AAV2/2(quad Y-F) serotype containing a double-floxed inverted transgene comprising one of the opsins of interest under a CMV promoter. None of the opsins investigated caused changes in retinal thickness; induced apoptosis in the retina or in transgene expressing cells; or reduced expression of PKCα (a specific bipolar cell marker). No increase in retinal inflammation at the level of gene expression (*IBA1/AIF1*) was found within the treated mice compared to controls. The expression of hRHO, CoChR or ReaChR under a strong constitutive promoter in retinal ON bipolar cells following intravitreal delivery via AAV2 does not cause either gross changes in retinal health, or have a measurable impact on the survival of targeted cells.

## 1. Introduction

Optogenetic strategies show promise as therapeutic approaches to restore photosensitivity in advanced retinal degeneration [1,2,3,4,5,6,7]. In conditions such as retinitis pigmentosa, neurons within the inner retina survive photoreceptor degeneration, making them suitable candidates for the ectopic expression of photosensitive proteins. An important consideration in the future development of these therapies is their safety. In particular, it is important to determine the impact of the therapeutic expression of optogenetic actuators on the cells in which they are expressed, and the wider retinal structure. On the one hand, there is the theoretical possibility that the ectopically expressed photosensitive protein may itself be toxic, or induce toxicity through means such as chronic activation [8]. On the other, toxicity may arise from other aspects associated with viral gene therapy, shown with recent evidence suggesting that the viral delivery of transgenes, including non-selective promoters, can be toxic in the retina [9].

Here, we set out to test the safety of the viral expression of human rod opsin (hRHO) and two channelrhodopsins; a *Chloromonas oogama channelrhodopsin mutant* (CoChR-3M) and red-shifted channelrhodopsin variant (ReaChR) in ON bipolar cells of the mouse retina. Multiple opsins have been shown to restore a functional basis of vision when ectopically expressed within the remaining intact retina of rodent models of retinal degeneration, including microbial light-gated ion channels and pumps [7], and naturally occurring and synthetic light-gated G-protein coupled receptors [2,6,10,11]. For this study, we firstly chose to investigate human rod opsin [3,12], as it is a native human protein and possesses advantages in terms of photosensitivity over standard microbial opsins [13,14]. To accompany the hRHO dataset, we also investigated two channelrhodopsins with enhanced therapeutic potential: CoChR-3M, which has enhanced light sensitivity [15]; and a red-activatable channelrhodopsin, ReaChR, which possesses improved light tissue penetrating properties compared to the normal shorter wavelength counterparts [16]. In all cases, we targeted opsin expression to ON bipolar cells, as several studies have reported that this strategy provides the best outcome in terms of visual restoration [4,6,12,17,18].

The delivery method of choice for recent optogenetic therapies are adeno associated viruses (AAVs), which are now applied in a clinical setting for retinal gene therapy [19]. Here, we employed a capsid mutant (quad Y-F) of the AAV2 serotype, which has been shown to provide good transduction of bipolar cells following intravitreal injection in the degenerate mouse retina [20,21]. In order to restrict the expression to ON bipolar cells, we employed a mouse line (*Grm6^cre^*) in which Cre recombinase is expressed in these cells [22]. The *GRM6* gene is responsible for encoding the metabotropic glutamate receptor mGluR6, which is localised within ON bipolar cells [23,24]. In this way, we hoped to achieve ON bipolar cell-specific transgene expression using Cre-dependent activation of a transgene comprising an opsin under the control of a strong ubiquitous promoter (CMV). This had several advantages over the alternative of employing an ON bipolar cell-specific promoter (e.g., grm6-SV40) in the viral transgene [17]. Firstly, it provided greater confidence that the transgene expression was restricted to the target cells. Secondly, it allowed us to use a strong, ubiquitous promoter for our transgene construct that has previously been shown to enhance toxicity, thus maximising the potential for our study to reveal safety concerns.

## 2. Results

### 2.1. Expression of Human Rod Opsin in ON Bipolar Cells Using the Grm6^cre^ Mouse

**Figure 1:**
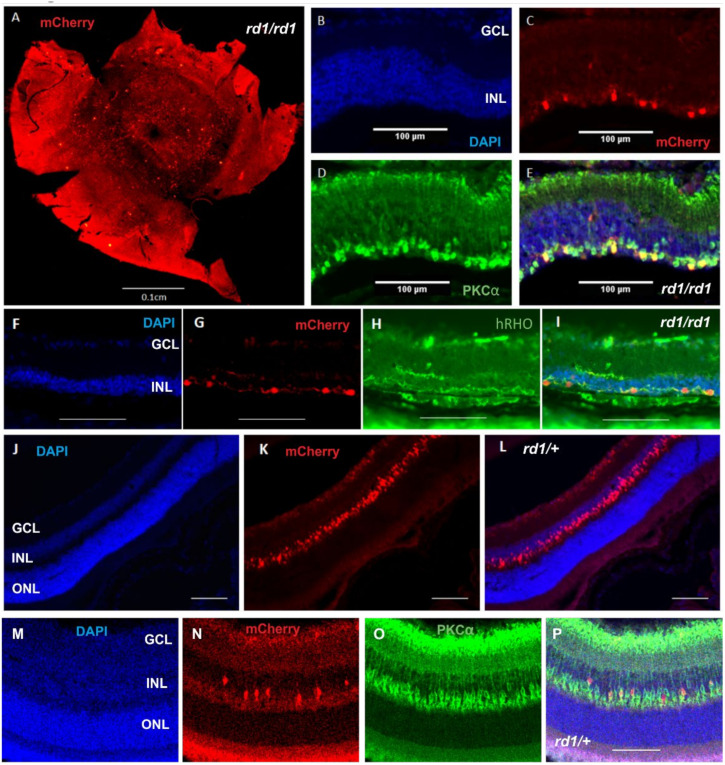
Characterisation of a *Grm6^Cre^ Rd1* mouse treated with human rod opsin. (**A**) A representative wholemount retinal image of a *Grm6^Cre/+^ rd1/rd1* mouse following an intravitreal injection of AAV2(4Y-F)-CMV-DIO-hRHO-T2A-mCherry displaying widespread transduction of the mCherry reporter. (**B**–**E**) Cross section of a rod opsin-treated retina of a *Grm6^Cr/+e^ rd1/rd1* mouse displaying mCherry co-localisation within PKCα-expressing bipolar cells. (**B**) DAPI (in blue), (**C**) mCherry (in red), (**D**) PKCα (in green), (**E**) merge of B, C and D. (**F**–**I**) Cross section of a rod opsin-treated *Grm6^Cre^ rd1/rd1* mouse displaying mCherry co-localisation with human rod opsin (hRHO). (**F**) DAPI (in blue), (**G**) mCherry (in red), (**H**) rod opsin (in green), (**I**) = merge of F, G and H. (**J**–**L**) A cross section of a rod opsin-treated retina of a *Grm6^Cre^ rd1/+* mouse. (**F**) DAPI (in blue), (**G**) mCherry (in red), (**H**) = merge of **F** and **G**. (**M**–**P**) Cross section of a rod opsin-treated *Grm6^Cre^ rd1/+* mouse displaying mCherry co-localisation with PKCα. (**M**) DAPI (in blue), (**N**) mCherry (in red), (**O**) PKCα (in green), (**P**) merge of M, N and O. Scale bars = 100 μm. ONL = outer nuclear layer; INL = inner nuclear layer; GCL = ganglion cell layer.

Intravitreal injections of DIO rod opsin–mCherry AAV2 to *rd1/rd1;Grm6^Cre^* mice resulted in transgene expression across the retina (Figure 1A). Examination of the retinal sections revealed mCherry expression in the outer portion of the inner nuclear layer (INL) in the degenerate retinas, as expected for ON bipolar cell targeting. Further to this, immunocytochemical staining for the ON bipolar cell marker PKCα revealed strong co-localisation with the mCherry reporter (Figure 1B–E), and that mCherry-expressing cells also co-localised with the rod opsin expression (Figure 1F–I). We also included the non-degenerate mice (*rd1/+;Grm6^Cre^*) in our study to ensure that any transgene-induced change in morphology was not masked by ongoing degeneration. This pattern of mCherry expression was recapitulated in the non-degenerate (*rd1/+;Grm6^Cre^*) retina (Figure 1J–P). These data confirm that the combination of a ubiquitous promoter in the DIO system with the *Grm6^Cre^* mouse represents an effective strategy for driving transgene expression in mouse ON bipolar cells.

### 2.2. Gross Retinal Anatomy Is Not Impaired by Viral Delivery of Rod Opsin to ON Bipolar Cells

As a first step to determine whether rod opsin treatment is detrimental to retinal integrity, we applied optical coherence tomography (OCT) to measure the retinal thickness in vivo. To maximise our ability to detect changes in retinal integrity, we established a longitudinal study in which we could assess changes in the retinal thickness over time in groups of injected and non-treated control mice, as well as in both retinally degenerate and non-degenerate mice. The mice were injected at 2 months of age (after a baseline OCT) and OCT measures were made at 3, 5 and 8 months old (1, 3 and 6 months post-treatment).

**Figure 2:**
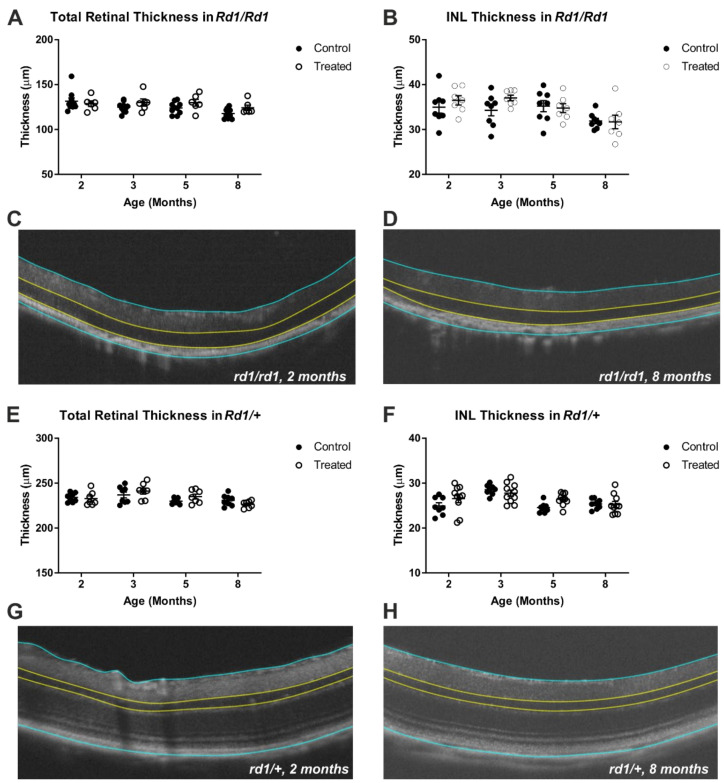
Longitudinal study of retinal thickness in a *Grm6^Cre^ Rd1* mouse treated with human rod opsin. Total retinal thickness (**A**) and INL thickness (**B**) in rod opsin-treated and control mice within degenerate retinas (control *n* = 8, treated *n* = 7). Representative OCTs of retinae pre-treatment at 2 months old (**C**) and post-rod opsin treatment at 8 months old (**D**) from *rd1/rd1*. Longitudinal study of total retinal thickness (**E**) and INL thickness (**F**) in rod opsin-treated and control mice within non-degenerate *rd1/+* retinas (control *n* = 8, treated *n* = 10). Representative OCTs of retinae pre-treatment at 2 months old (**C**) and post-rod opsin treatment at 8 months old (**D**) from *rd1/rd1* and at 2 months old (**G**) and post-rod opsin treatment at 8 months old (**H**) in *rd1/+*. No significant difference was found between the control and treated groups in either group; however, time was shown to have a significant effect on INL thickness (*p* = 0.0037 (*rd1/rd1*), *p* < 0.0001 (*rd1/+*)) and total retinal thickness (*p* = 0.0175 (*rd1/rd1*), *p* = 0.0004 (*rd1/+*)) (two-way mixed ANOVA, repeated measures, Bonferonni multiple).

We found no evidence of retinal thinning associated with the gene therapy in either retinally degenerate or intact animals at any of the timepoints tested. As expected, both the inner nuclear layer (INL) and, to a more dramatic extent, the total retinal thickness were reduced in the retinally degenerate animals. The total retinal thickness decreased over time in both genotypes (two-way RM-ANOVA main effect of time (F = 3.767, *p* = 0.0394) for *rd1/rd1* and (F = 7.598, *p* = 0.001) for *rd1/+*) (Figure 2A,E), but there was no impact of treatment (main effect of treatment (F = 3.007, *p* = 0.1049) for *rd1/rd1* and (F = 0.1895, *p* = 0.6704) for *rd1/+*) and interaction (F = 1.209, *p* = 0.3182) for *rd1/rd1* and (F = 2.156, *p* = 0.1087) for *rd1/+*). A similar pattern was observed in INL thickness, with a significant decrease found over time in both genotypes (two-way mixed ANOVA main effect of time (F = 5.287, *p* = 0.0037) for *rd1/rd1* and (F = 9.842, *p* < 0.0001) for *rd1/+*), also with no impact of treatment (main effect of treatment (F = 1.451, *p* = 0.2498) for *rd1/rd1* and (F = 1.458, *p* = 0.2448) for *rd1/+* and interaction (F = 0.8602, *p* = 0.4699) for *rd1/rd1* and (F = 2.365, *p* = 0.0826) for *rd1/+*) (Figure 2B,F). Representative images of the retinal thickness assessments from the OCTs are shown from a pre-injection timepoint (2 months old) and the last assessment (8 months old) in retinally degenerate (Figure 2C,D, respectively) and non retinally degenerate (Figure 2G,H, respectively) mice. Within these images, the blue lines represent the borders used to measure the total retinal thickness, and the yellow lines represent the borders for the INL measurement.

### 2.3. ON Bipolar Cell Survival Is Not Impacted by Viral Delivery of Rod Opsin

**Figure 3:**
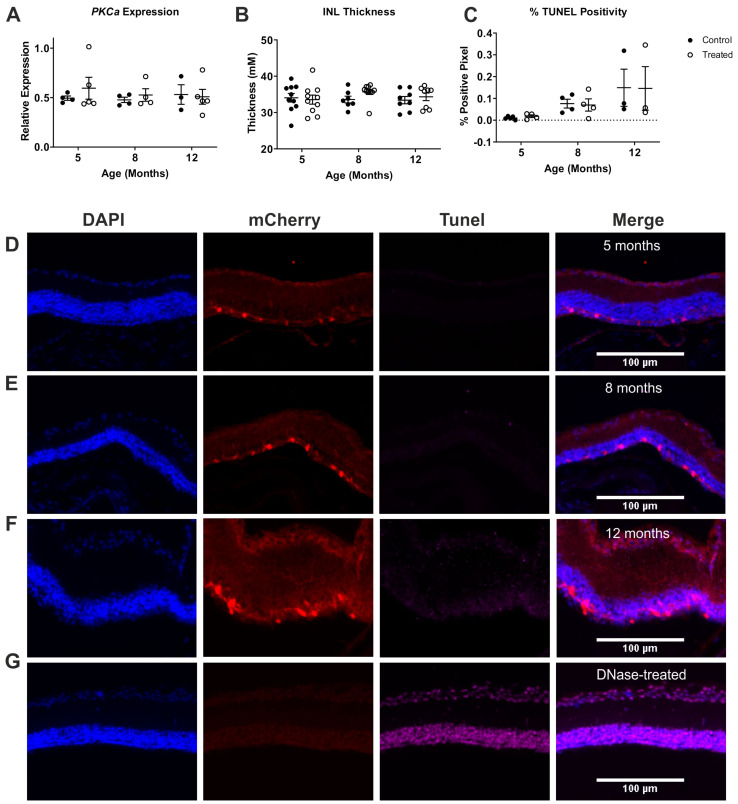
Toxicity study on a cellular level of rod opsin-treated *Grm6^Cre^ rd1/rd1* retinas. (**A**) PKCα expression in rod opsin-treated and control *rd1/rd1* mice. (**B**) INL thickness of rod opsin-treated and non-treated control *rd1/rd1* mice (**C**) Levels of apoptosis (TUNEL positivity) within the INL of rod opsin-treated and control *rd1/rd1* mice. (**D**–**F**) Representative images of mCherry and TUNEL co-localisation at 5 months of age (**D**), 8 months of age (**E**), 12 months of age (**F**) and a DNase-treated positive control (**G**). No effect of the treatment was found on the INL thickness (two-way mixed ANOVA *p* > 0.05); or on the *PKCa* expression (two-way mixed ANOVA *p* > 0.05). Across the INL, the positive pixel count was always low (< 0.4%), but this did increase with age, and time was shown to have a significant effect (two-way mixed ANOVA, *p* < 0.05). Importantly, however, the levels of apoptosis were not impacted by treatment (two-way ANOVA, *p* > 0.05).

Next, we asked whether the ON bipolar cell population in particular was impacted by our viral gene delivery of rod opsin in to *rd1/rd1;Grm6^Cre^* mice. Our first approach was to assess the level of expression of an ON bipolar cell marker (*PKCa*) at 3, 6 and 10 months post-injection with the viral vector (5, 8 and 12 months of age). Prior to collecting tissue for this study, we measured OCTs to provide a context for our findings. Consistent with the results of our longitudinal study, we found no evidence of INL thinning induced by the treatment (Figure 3B, two-way mixed ANOVA main effect of time (F = 0.3579, *p* = 0.7011), treatment (F = 0.9100, *p* = 0.3450) and interaction (F = 0.7847, *p* = 0.4622)). Turning to *PKCa* expression, qPCR from the whole retinas revealed equivalent levels of this ON bipolar cell marker in the treated and untreated groups across the time points tested (Figure 3A, two-way mixed ANOVA main effect of time (F = 0.1562, *p* = 0.8564), treatment (F = 0.4298, *p* = 0.5199) and interaction (F = 0.3066, *p* = 0.7395)).

As a final test of toxicity, we took the other eye from this group of animals and subjected it to TUNEL staining to reveal apoptotic cells. An exploration of the retinal sections revealed that the levels of TUNEL staining were very low across all three investigated time points in our treated groups, and significantly lower than in a positive control section treated with DNAse (Figure 3G). This impression was confirmed by the quantification of TUNEL staining (positive pixel count) (Figure 3C). Across the INL, the positive pixel count was always low at < 0.4%, but this did increase with age, and time was shown to have a significant effect (two-way mixed ANOVA, F = 4.583, *p* = 0.0256). Importantly, however, the levels of apoptosis were not impacted by treatment (two-way mixed ANOVA main effect of treatment (F = 0.0002, *p* = 0.9885) and interaction (F = 0.0165, *p* = 0.9836)). A possibility is that TUNEL staining could be higher in the fraction of INL neurones expressing the transgene. In practice, this proved not to be the case. Thus, out of 453 mCherry stained cells (from all ages), we found only 2 that had TUNEL-positive nuclei.

### 2.4. Expression of Channelrhodopsin Variants Does Not Impact Gross Retinal Anatomy or Bipolar Cell Number

**Figure 4:**
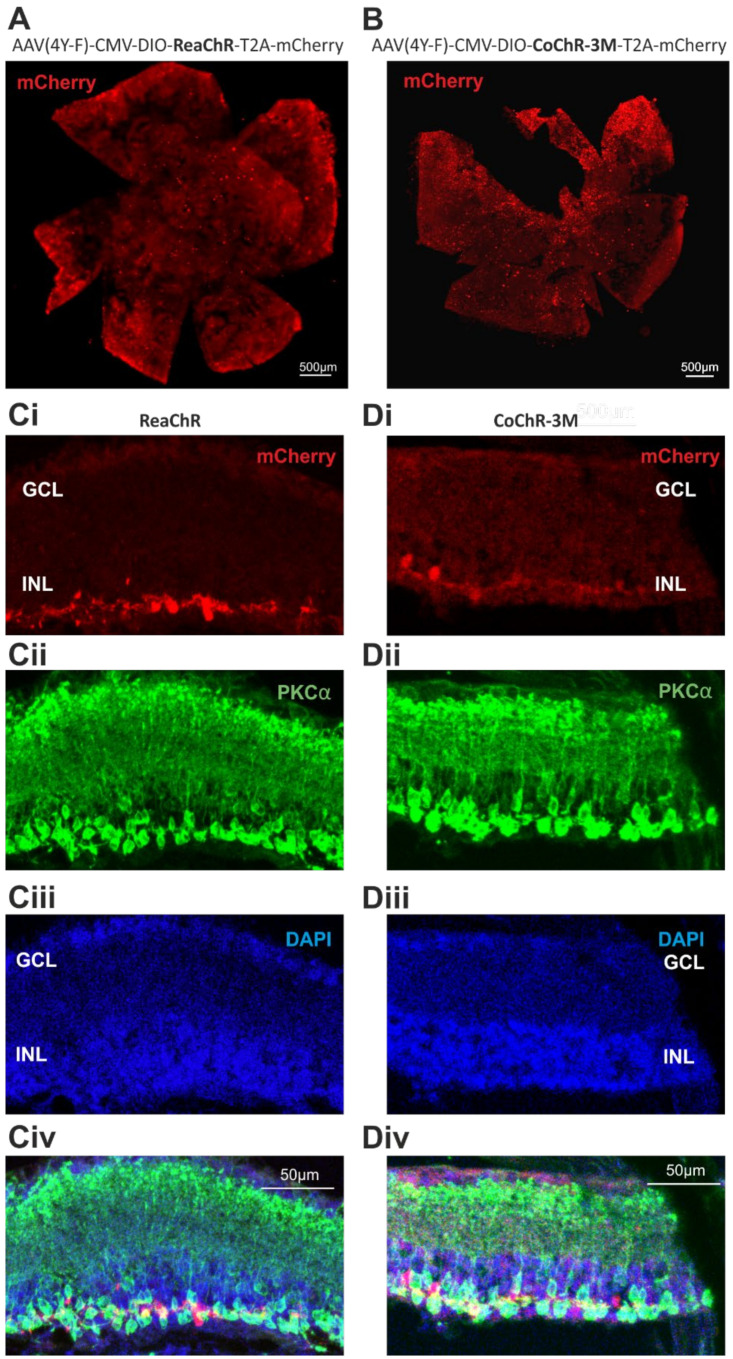
Characterisation of a *Grm6^Cre^ rd1* mouse treated with channelrhodopsins: ReaChR and CoChR-3M. (**A**,**B**) Representative retinal wholemount images of *Grm6^Cre^ rd1/rd1* mice following intravitreal injection of ReaChR in (**A**) and CoChR-3M in (**B**), displaying widespread transduction of the mCherry reporter. (**C**(**i**–**iv**),**D**(**i**–**iv**)) Retinal cross sections of *Grm6^Cre^ rd1/rd1* mice following intravitreal injection of ReaChR and CoChR-3M in (**B**) displaying mCherry co-localisation within PKCα-expressing bipolar cells: ReaChR and CoChR-3M. (**C**(**i**),**D**(**i**)) mCherry (in red); (**C**(**ii**),**D**(**ii**)) PKCα (in green) and (**C**(**iii**),**D**(**iii**)) DAPI (in blue), (**C**(**iv**),**D**(**iv**)) represent merge of corresponding images. ONL = outer nuclear layer; INL = inner nuclear layer; GCL = ganglion cell layer.

Next, we applied a similar approach to investigate the safety of two channelrhodopsins with promising characteristics for vision restoration, a *Chloromonas oogama* more light-sensitive channelrhodopsin mutant (CoChR-3M), and a red-sensitive channelrhodopsin (ReaChR). The viral gene delivery of these opsins with an mCherry reporter (vector maps shown in Supplementary Figure S1) in *rd1/rd1;Grm6^Cre^* mice resulted in the widespread distribution of mCherry expression throughout the wholemounts (Figure 4A–C), comparable to that of rod opsin-treated mice (Figure 1A). Co-localisation with PKCa confirmed transgene expression in ON bipolar cells within the retinal cross sections (Figure 4D–G). Some mCherry positive cells were found to be PKCa-negative, but as these are located in INL, they may be cone ON bipolar cells [17].

**Figure 5:**
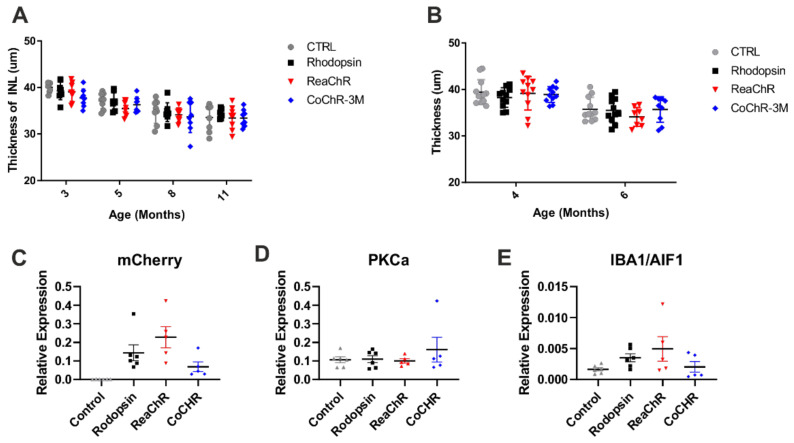
Toxicity study of opsin-treated *Grm6^Cre^ rd1/rd1* retinae. (**A**) Longitudinal study and (**B**) non-longitudinal study of INL thickness in rod opsin; ReaChR- and CoChR-3M-treated and control mice. (**C**) mCherry relative expression in rod opsin; ReaChR- and CoChR-3M-treated and non-injected control mice. (**D**) *PKCα* relative expression and (**E**) *IBA1/AIF1* relative expression in rod opsin; ReaChR- and CoChR-3M-treated and control mice. There was a significant decrease in the INL thickness observed over time in all mice (two-way RM-ANOVA main effect of time *p* < 0.001), but no impact of any of the treatments was found (*p* > 0.05). Analysis of the expression of *PKCa* and *IBA1/AIF1* found no difference between any of the treatments compared to controls (the Kruskal–Wallis test with Dunn’s multiple comparisons correction, *p* > 0.05).

We undertook a longitudinal investigation into retinal thickness using OCT in cohorts of *rd1/rd1;Grm6^Cre^* mice expressing either one of the two channelrhodopsins or, for comparison, rod opsin or non-injected control mice. The mice were injected between 60 and 80 days and OCTs were recorded at 1, 3, 6 and 9 months post-treatment (3, 5, 8 and 11 months of age). There was a significant decrease in the INL thickness observed over time in all mice (two-way RM-ANOVA main effect of time F = 61.89, *p* < 0.001), but no impact of any of the treatments was found (two-way RM-ANOVA main effect of treatment (F = 1.307, *p* = 0.2918) or interaction (F = 0.6868, *p* = 0.7189)) (Figure 5A).

To identify the possible impacts on bipolar cell survival and retinal inflammation, a further cohort of animals was injected with a virus and scheduled for qPCR analysis of *PKCa* and *IBA1/AIF1* expression at 4 months post-treatment. The OCT measurements at 2 and 4 months after treatment confirmed that there was no impact from any of the treatments (two-way mixed ANOVA main effect of treatment (F = 0.5861, *p* = 0.6260) or interaction (F = 0.7401, *p* = 0.5314)) (Figure 5B). At 4 months, the qPCR analysis of mCherry expression (normalised to GADPH) revealed comparable levels of expression for the three transgenes compared to the control (Figure 5C; the Kruskal–Wallis test with Dunn’s multiple comparisons correction, *p* = 0.9789). The analysis of the expression of *PKCa* and *IBA1/AIF1* found no difference between any of the treatments compared to controls (Figure 5D,E; the Kruskal–Wallis test with Dunn’s multiple comparisons correction, *p* = 0.2051). These results suggest that there was no change in the rod ON bipolar cell number or retinal inflammation at the level of gene *IBA1/AIF1 expression* due to the opsin treatments.

## 3. Discussion

Our study did not reveal significant safety concerns for optogenetic therapies involving hRHO or the two channelrhodopsins investigated (*CoChR-3M, and ReaChR)* expressed in ON bipolar cells. We combined a transgenic mouse (*Grm6^Cre^ rd1/rd1*) with intravitreal injection of a recombinant AAV2(4Y-F) vector providing Cre-dependent expression of the opsin of interest under a strong constitutive promoter (CMV) to introduce expression into ON bipolar cells. We showed that this strategy produced transgene expression in the target cells, and that no evidence of detrimental effects on retinal integrity linked to the transgene was found using a variety of approaches.

OCT measurements of retinal thickness are commonly used in human and animal studies to track retinal degeneration. This method has the ability to reveal very small changes in retinal thickness [25] and can reveal both swelling and thinning of the retina associated with many disease states. Importantly, because this method is non-invasive, it can be applied in a repeated measures design to track changes in individual mice over time, enhancing statistical power. We applied this approach here to explore the long-term effects of the optogenetic therapy. We found no change in thickness associated with treatment in either degenerate or intact retinas, which suggests that there was no effect on the retinal tissue that would be masked by ongoing degeneration. This was true whether we measured the whole retina, or concentrated on the region targeted for expression (the inner nuclear layer). We also found no changes in the INL thickness of mice treated with CoChR-3M and ReaChR.

The OCT data thus confirm that at a gross level, the retinas in *rd1/rd1;Grm6^Cre^* mice are not affected, even up to 9 months after treatment, compared to controls. A legitimate concern is that toxicity in the cells actually expressing the transgene may not be apparent in such measures of the whole retinal population. We addressed that possibility first by looking for changes in the expression of an ON bipolar cell-specific marker. Such an approach has been used to reveal ganglion cell degeneration following oxygen/glucose deprivation (by measuring the ganglion cell marker *THY1*) [26]. In principle, this should also be applicable to the assessment of bipolar cells with the use of *PKCa* (a specific marker of rod bipolar cells (RBCs) [27,28]). Previous studies have shown a decrease in the *PKCa* expression at the protein level as a result of NMDA excitotoxicity, which was correlated to a decrease in the health/functional state of RBCs [29]. Here, we found no evidence of a decrease in *PKCa* expression at the RNA level in hRHO-, CoChR-3M-, or ReaChR-treated mice. Further to this, we also investigated *IBA1/AIF1* expression as a means of assessing any potential changes in retinal inflammation due to treatment [30]. No increases in *IBA1/AIF1* expression were found with any treatment when assessed as individual treatments vs. control, or as a grouped assessment (all treatments grouped vs. control), indicating that the treatment and delivery method induced no significant increase in retinal inflammation at the level of *IBA1* gene expression compared to the non-treated controls. Future studies will be needed to further assess potential inflammation by investigating the protein expression of AIF1 and glial fibrillary acidic protein (GFAP), as well as activation of Müller cells staining.

As viral transduction can never successfully target all ON bipolar cells across the whole retina, we finally looked for evidence of degeneration specifically in those neurons that actually expressed the transgene. Our approach was to use TUNEL staining at early, mid and late time points after injection to determine whether there was evidence of apoptosis in transgene-expressing neurons. Consistent with our other findings, TUNEL staining was very low in the retinas of treated animals (and not significantly enhanced compared to controls). Moreover, there was no indication of enhanced TUNEL staining in the INL as a whole, nor in the hRHO cells expressing the transgene.

The data presented here provide reassurance that a therapy based upon rod opsin or investigated channelrhodopsins need not have substantial safety concerns. One issue that we have not addressed is the possibility that the long-term exposure to the high light levels required for the optogenetic activation of microbial opsins might have detrimental effects. Our study, however, did include rod opsin and Co-ChR-3M, which are both activated at moderate light intensities and would have experienced optogenetic activation. The mice treated with these opsins displayed no evidence of retinal degeneration, indicating that moderate light intensity and optogenetic activation of the likes of hRHO and Co-ChR-3M induce no additional retinal degeneration in comparison to opsins that will not have seen optogenetic activation, such as ReaChR. A more significant limitation of our study is that, in mice, we cannot replicate the very long treatment times (many years) that would be required for a human therapy. Nevertheless, within the constraints of a rodent study, we have tried to replicate long-term expression (up to 9 months post-injection) and found no evidence of retinal degeneration due to the opsin expression. An important finding from this study is that we found no link between the ubiquitous promoter (CMV) and bipolar cell death. This issue was highlighted in a recent study that showed that the promoter used for transgene expression can have a substantial impact on toxicity in ocular gene therapy [9]. We found no evidence of bipolar cell-linked toxicity with the use of this ubiquitous promoter. Future studies will be needed to address any potential safety concerns with the specific promoters designed to allow targeted gene delivery in humans [31].

## 4. Methods

### 4.1. Mice

All animal experiments were conducted in accordance with the UK Animals (Scientific Procedures) Act (1986) and approved by the UK Home Office under the animal licence PP3176367. Adult *Grm6^Cre^* mice [22] were a kind gift from Robert Duvoisin (Oregon Health Sciences) and kept on a mixed background of C57BL/6-C3H/HeH, where they were bred to be non-retinally degenerate (*rd1/+)* or retinally degenerate (r*d1/rd1*). The mice were intravitreally injected in both eyes with 2.5 µL of AAV2(4Y-F) viral vectors containing one of the opsins (Vectorbuilder, vector maps shown in Supplementary Figure S1) between the ages of 60 and 80 days. The mice were culled via cervical dislocation at designated endpoints, in the case of the non-longitudinal samples at time points 3, 5, 8 and 12 months of age for the hRHO study, and at 6 months of age for the channelrhodopsin study. In the case of the longitudinal study, all eyes were retrieved at 8 months of age for the hRHO study.

### 4.2. Immunohistochemistry

The eyes were collected and fixed in 4% paraformaldehyde (PFA) in PBS overnight. After fixing, the eyes were then placed into 30% sucrose in PBS overnight for cryopreservation. The eyes were embedded in OCT and frozen on dry ice before being sectioned at 20 µM thickness.

For mCherry staining, sections were blocked in blocking solution (5% donkey serum in PBS with 0.1% Triton X) before the application of rabbit polyclonal anti-mCherry antibody (Kerafast, USA) to the slides at 1:400 in blocking solution at 4 °C overnight. After washing, donkey anti-rabbit Alexa 546 (Invitrogen, 1:500) was applied to the slides for 2 h at room temperature. Further washing then took place before the addition of either monoclonal rabbit anti-PKCa (Abcam, 1:500), or monoclonal mouse anti-rhodopsin (4D2) (Abcam, 1:500) was applied to the slides in blocking solution overnight at 4 °C. After washing, donkey anti-rabbit or mouse Alexa 488 (Abcam, 1:500) was applied to the slides for 2 h at room temperature. The slides were mounted with ProLong Gold Antifade with DAPI (ThermoFisher, UK) and allowed to dry overnight. For mCherry and PKCa co-staining, following the staining for mCherry, the slides were washed for 2 h longer and then blocked again in blocking solution (5% donkey serum in PBS with 0.1% Triton X). Further washing then took place before monoclonal rabbit anti-PKCa (Abcam, 1:400) was applied to the slides in blocking solution overnight at 4 °C. After washing, donkey anti-rabbit Alexa 647 (Invitrogen, 1:500) was applied to the slides for 2 h at room temperature. The slides were mounted with ProLong Gold Antifade with DAPI (ThermoFisher) and allowed to dry overnight.

Apoptotic cells were detected utilising the Click IT Plus TUNEL assay tagged with Alexa Flour 647 (ThermoFisher), according to the manufacturer’s instructions.

The retinal wholemounts were dissected after the eyes’ fixation in 4% PFA overnight. The retinal wholemounts were then permeabilised in 1% Triton X in PBS for 1 h at room temperature, and then put into blocking solution that contained 10% donkey serum in 1% Triton X in PBS for 3 h at room temperature. The primary antibody rabbit polyclonal anti-mCherry antibody (Kerafast) was applied to the retinas at 1:400 in blocking solution at 4 °C overnight. The retinas were then washed in 0.2% Triton X in PBS and incubated with secondary antibody donkey anti-rabbit Alexa 546 (Invitrogen, 1:500), which was applied in blocking solution overnight at 4 °C. After sufficient washing of the retinas in 0.2% Triton X in PBS, they were mounted with fluorescent mounting media (ProLong Gold, ThermoFisher).

Images of the retinal cross sections with rod opsin therapy were collected on a Zeiss Axioimager D2 upright microscope using a 20 x/0.5 EC Plan-Neofluar objective and were captured using a Coolsnap HQ2 camera (Photometrics) through Micromanager software v1.4.23. Specific band pass filter sets for DAPI, FITC and Texas red were used to prevent bleed through from one channel to the next. Images of the retinal wholemounts were collected on a Leica M205 FA upright stereomicroscope using a 5 x/0.50 PlanAPO LWD objective at the equivalent of 10 × magnification and were captured using a DFC 365FX (Leica) camera through LAS AF v3.1.0.8587 software (Leica). Specific band pass filter sets for mCherry were used to prevent bleed through from one channel to the next. Images of the retinal cross sections from the channelrhodopsin therapies were collected on a Leica SP8x AOBS inverted confocal using a 40 x/0.85 HCX PL APO objective. The confocal settings were as follows: pinhole (1 airy unit), scan speed (600Hz bidirectional), format (1024 × 1024). The images were collected using (hybrid) detectors with the following detection mirror settings: DAPI 360–400 nm; Red 560–600 nm; and Cy5 640–690 nm, using the UV and white light lasers. When acquiring 3D optical stacks, the confocal software was used to determine the optimal number of Z sections. Only the maximum intensity projections of these 3D stacks are shown in the results.

The assessment of TUNEL staining was done in ImageJ and conducted using the following method: A DNase positive control sample was run alongside each group and the threshold of analysis set to 100%, whereby the number of TUNEL-stained pixels within the inner nuclear layer (INL) is equal to the number of pixels of DAPI-stained nuclei. This threshold was then applied to the treated and untreated samples and a percentage of apoptotic cell pixels to cell nuclei pixels was calculated.

### 4.3. PCR

The eyes were collected at designated endpoints and the retinae isolated and stored in RNAlater (ThermoFisher) until the RNA was collected using the RNeasy Mini Kit (Qiagen, UK), and cDNA produced using the Tetro cDNA synthesis kit (Bioline, UK). The gene expression was assessed using the LightCycler 480 (Roche, UK) with the LightCycler 480 SYBR Green Mastermix (Roche, UK). The gene expression for the hRHO studies was normalised via a relative expression method, whereby the hRHO was divided by the geometric mean of the three housekeeping genes ARP (acidic ribosomal phosphoprotein P0), GAPDH (glyceraldehyde-3-phosphate dehydrogenase) and PSMB2 (proteasome subunit beta type-2) [32]. For the channelrhodopsins and rod opsin comparison study, the gene expression was normalised to GAPDH. The primers were produced by Sigma (see Table 1).

### 4.4. Optical Coherence Tomography

The mice were anaesthetised using isoflurane and the retinal thickness assessed within the central region of the retina ventral to the optic nerve using the Phoenix Micron 4 system (Phoenix Technology Group, CA, USA). The retinal thickness was calculated using InSight (Phoenix Technology Group, CA, USA) version 2.0.5940. As part of the inclusion criteria for the OCT analysis, qPCR was conducted on all samples at the end of the study to confirm transgene expression within the treated samples and absence in the control samples.

## Figures and Tables

**Table 1:** List of primers used for qPCR.

ARP Fwd	CGACCTGGAAGTCCAACTAC
ARP Rev	ATCTGCTGCATCTGCTTG
GAPDH Fwd	TGCACCACCAACTGCTTAG
GAPDH Rev	GATGCAGGGATGATGTTC
PSMB2 Fwd	AAATGCGGAATGGATATGAATTG
PSMB2 Rev	GAAGACAGTCAGCCAGGTT
mCherry Fwd	GATAACATGGCCATCATCAAGGA
mCherry Rev	CGTGGCCGTTCACGGAG
PKCa/PRKCA Fwd	CGGAAGCCCTACCTTCTGTG
PKCa/PRKCA Rev	TCTCGTACCGTGACGTGGAG
hRHO Fwd	CCTGCTCAACCTAGCCGTG
hRHO Rev	AGGGCAATTTCACCGCCCA
IBA1/AIF1 Fwd	CAGACTGCCAGCCTAAGACA
IBA1/AIF1 Rev	AGGAATTGCTTGTTGATCCC

## Data Availability

Data available on request.

## Supplementary Materials

The following are available online at https://www.mdpi.com/article/10.3390/ijms222313111/s1.
