# Supplementary figures and images for: Viral Transduction of Human Rod Opsin or Channelrhodopsin Variants to Mouse ON Bipolar Cells Does Not Impact Retinal Anatomy or Cause Measurable Death in the Targeted Cells
Source: Int J Mol Sci. 2021 Dec 3;22(23):13111. doi: 10.3390/ijms222313111 (PMC8658283; doi:10.3390/ijms222313111)

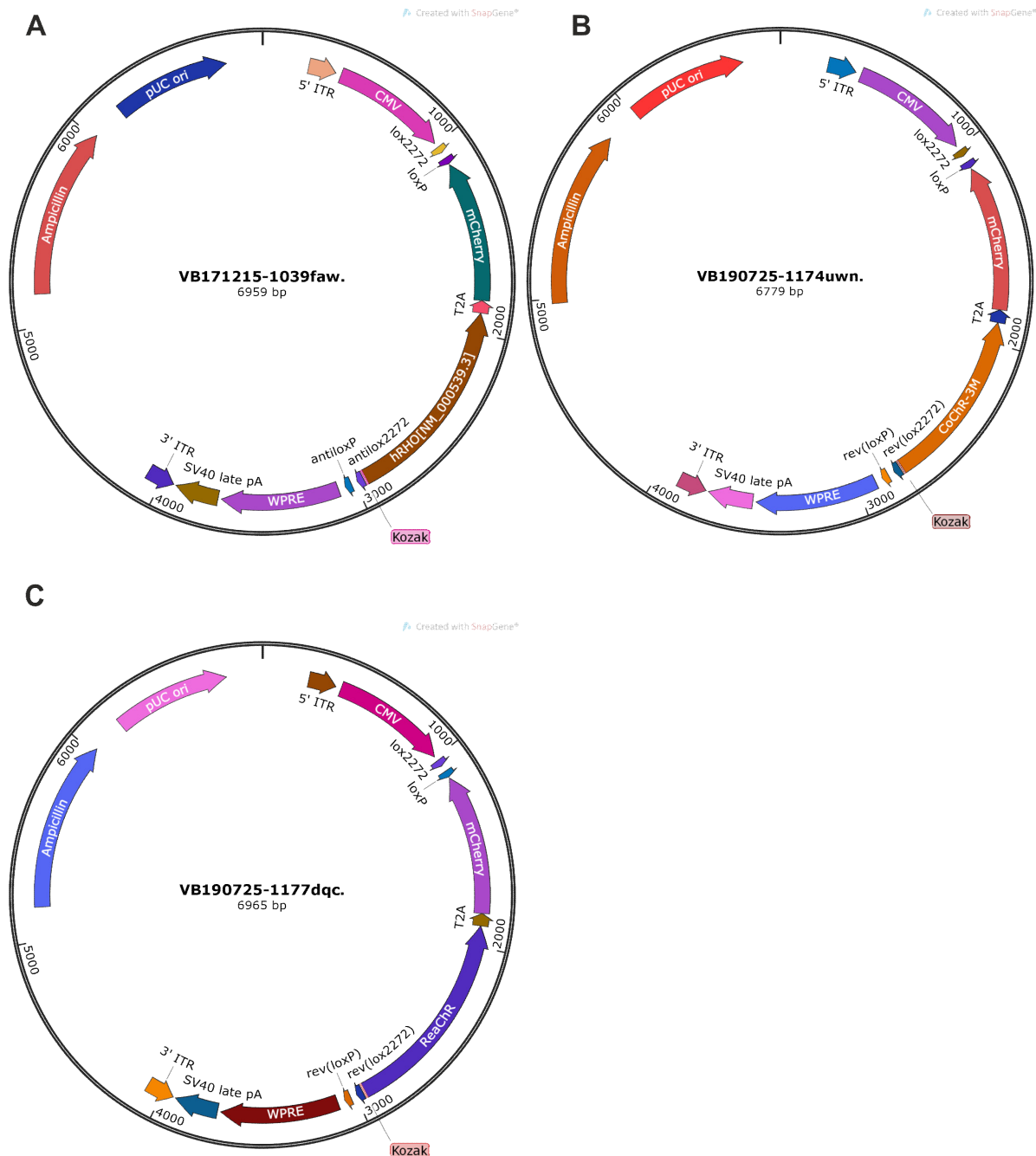

**Supplementary Figure S1.** Viral vector maps of human Rod opsin (A), CoChR-3M (B), ReaChR (C).

Supplement: Supplementary file 1 [file ijms-22-13111-s001.zip › ijms-1463026-supplementary.pdf]
